# Physicochemical and biological characterization of chitosan-microRNA nanocomplexes for gene delivery to MCF-7 breast cancer cells

**DOI:** 10.1038/srep13567

**Published:** 2015-09-01

**Authors:** B. Santos-Carballal, L. J. Aaldering, M. Ritzefeld, S. Pereira, N. Sewald, B. M. Moerschbacher, M. Götte, F. M. Goycoolea

**Affiliations:** 1 Institute of Plant Biology and Biotechnology (IBBP), University of Münster, Schlossgarten 3, D-48149 Münster, Germany; 2 Organic and Bioorganic Chemistry, Bielefeld University, Universitätsstrasse 25, D-33615 Bielefeld, Germany; 3 Department of Gynecology and Obstetrics, University of Münster, Albert-Schweitzer-Campus 1, D-48149 Münster, Germany

## Abstract

Cancer gene therapy requires the design of non-viral vectors that carry genetic material and selectively deliver it with minimal toxicity. Non-viral vectors based on cationic natural polymers can form electrostatic complexes with negatively-charged polynucleotides such as microRNAs (miRNAs). Here we investigated the physicochemical/biophysical properties of chitosan–hsa-miRNA-145 (CS–miRNA) nanocomplexes and the biological responses of MCF-7 breast cancer cells cultured *in vitro*. Self-assembled CS–miRNA nanocomplexes were produced with a range of (+/−) charge ratios (from 0.6 to 8) using chitosans with various degrees of acetylation and molecular weight. The Z-average particle diameter of the complexes was <200 nm. The surface charge increased with increasing amount of chitosan. We observed that chitosan induces the base-stacking of miRNA in a concentration dependent manner. Surface plasmon resonance spectroscopy shows that complexes formed by low degree of acetylation chitosans are highly stable, regardless of the molecular weight. We found no evidence that these complexes were cytotoxic towards MCF-7 cells. Furthermore, CS–miRNA nanocomplexes with degree of acetylation 12% and 29% were biologically active, showing successful downregulation of target mRNA expression in MCF-7 cells. Our data, therefore, shows that CS–miRNA complexes offer a promising non-viral platform for breast cancer gene therapy.

Cancer is the consequence of uncontrolled cell proliferation and the dysregulation of cellular processes such as differentiation and programmed cell death^1^. Most cancers are caused by mutations or chromosome rearrangements that directly affect the expression of oncogenes or tumour-suppressor genes, but there is increasing evidence that cancer can be triggered by changes in gene regulation, including the expression of small non-coding RNA molecules known as microRNAs (miRNAs)^2^,^3^.

MicroRNAs are small endogenous RNAs, approximately 22 nucleotides in length, which regulate eukaryotic gene expression on the post-transcriptional level. Only a small number of human miRNAs have been functionally characterized and many of these regulate cancer-related processes such as cell growth and differentiation, and therefore, potentially function as oncogenes. Expression profiling using miRNAs rather than protein-coding genes provides a more accurate method for the classification of cancer subtypes^2^,^4^. The differential expression of certain miRNAs in tumours is, therefore, a powerful tool for the diagnosis and treatment of cancer.

The introduction of miRNAs into human cells could provide an efficacious therapeutic approach to inhibit tumour progression^5^. However, successful gene therapy requires the development of suitable vehicles for the efficient delivery of nucleic acids to specific target cells, with minimal toxicity. Both viral and non-viral vectors have been developed for gene therapy. Viral vectors have been designed based on a wide range of viruses and typically include strong promoters that achieve a high level of heterologous gene expression. However, the use of viral vectors in human clinical trials raises significant safety issues, such as potential immunogenicity and reversion to pathogenicity^6^,^7^. This has encouraged the development of non-viral vectors with better biosafety profiles, including nanocomplexes based on dendrimers^8^, lipids,^9^ or polysaccharides^10^.

Non-viral transfection reagents composed of dendrimers (e.g. polyethylenimine) and lipid formulations (e.g. Lipofectamine) are already commercially available, but research has focused more recently on biocompatible vectors suitable for *in vivo* therapeutic use, including cationic polymers such as polysaccharides that form complexes with negatively charged polynucleotides (DNA and various forms of RNA). These polycations are conceived as building blocks that stabilize the genetic material, protect it from degradation, promote its uptake into target cells, and then efficiently unpack and deliver the nucleic acids^6^.

Chitosans (CS) are a family of linear, cationic heteropolysaccharides produced by the partial deacetylation of chitin, which is isolated from crustacean shells. They are biodegradable polymers composed of randomly distributed *β*(1 → 4)-linked *N*-acetyl-d-glucosamine and d-glucosamine units. The cationic properties of chitosan are useful in pharmaceutical formulations and biomaterials because the molecule can form polyelectrolyte complexes (also known as polyplexes) with plasmid DNA and, as recently reported, also with short interfering RNA (siRNA), making it an attractive delivery system^10^,^11^,^12^. Thus far, few studies have systematically investigated how the structure of chitosans, specifically the degree of acetylation (DA), pattern of acetylation (PA), and degree of polymerization (DP), affects the biophysical characteristics and biological functionality of chitosan-based polyplexes. Indeed, attempts have been made to establish a relationship between the DP and DA of chitosan, its salt form and pH on the efficiency of transfection with plasmid DNA *in vitro*^13^,^14^,^15^,^16^,^17^ and to determine the intracellular trafficking routes underlying their mode of action^18^. An ideal balance between the strength of the interaction between chitosan and plasmid DNA and the dissolution of the complex within the cell (thus conferring optimal transfection efficiency) can be achieved using chitosan molecules with specific DPs and DAs^19^. The biophysical properties of chitosan–siRNA complexes and their capacity for transfection have been investigated in detail^20^ and beneficial properties include a low molecular weight, a high DA, a small particle size (100 nm), and a moderate positive surface zeta potential along with a high (+/−) charge ratio^21^. However, it is unclear how these factors contribute to the observed transfection efficiency and further investigation is required. The delivery of microRNAs has been tested with functionalized gold nanoparticles, combined with either the stem-loop or hairpin structure of a miRNA^22^,^23^. The properties of chitosan–miRNA (CS–miRNA) complexes have not been documented.

Here we report a comprehensive investigation of the structure–function relationship in CS–miRNA systems, to determine whether the physicochemical and biophysical properties of different chitosans and miRNAs are related (e.g. size, zeta potential, chemical affinity of interaction and conformation). We also studied the ease with which miRNAs are released from the CS–miRNA complexes and subsequently processed within the cell to achieve the intended biological activity.

## Results

### Characterization of chitosans

The parent chitosan was depolymerized using two different amounts of nitrous acid (calculated according to the stoichiometry of the reaction) to obtain high and low molecular weight chitosans, here denoted as HDP and LDP, respectively. This is a selective reaction in which the nitrosating species attack the amine groups and cleave *β*-glycosidic but not *N*-acetyl linkages^24^,^25^. Chitosans with four different DAs were obtained by the acetylation of unprotonated primary amino groups with acetic anhydride. The DA of the chitosans was evaluated by ^1^H-NMR as previously described^26^,^27^,^28^. The viscosity average molecular weight (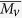) was determined from the intrinsic viscosity values using the corresponding Mark-Houwink constants^29^ (Table 1).

### Hybridization reaction

**Figure 1:**
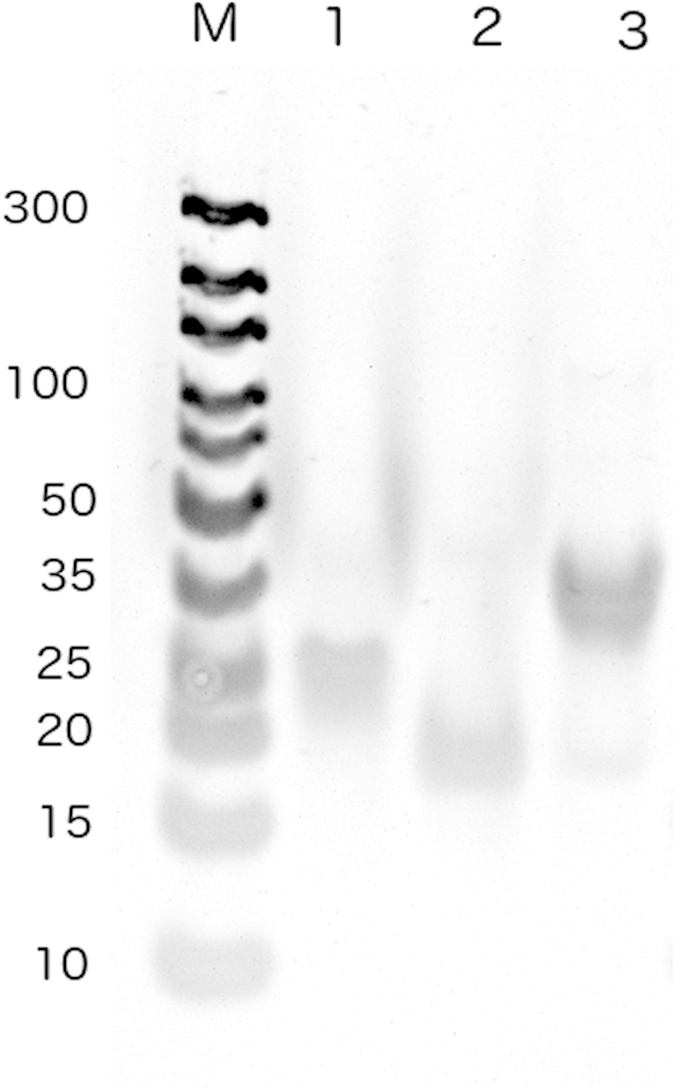
Polyacrylamide gel electrophoresis (15%) using 1x TAE buffer (constant voltage of 120 V, 90 min) and stained with SYBR gold (1x). Lane codes: M. Generuler ultra low range DNA ladder; 1. hsa-miR-145-3p; 2. hsa-miR-145-5p; 3. miRNA-145.

Analysis of the annealing reaction by gel electrophoresis (Fig. 1) revealed unique bands for both single-stranded miRNAs, comparable with the 20-mer to 25-mer size marker. The formation of the double stranded (duplex) was confirmed by the reduced mobility of the corresponding band reflecting its higher molecular mass (14.2 kDa) compared to the single strands.

### Physicochemical characterization of CS–miRNA complexes

**Figure 2:**
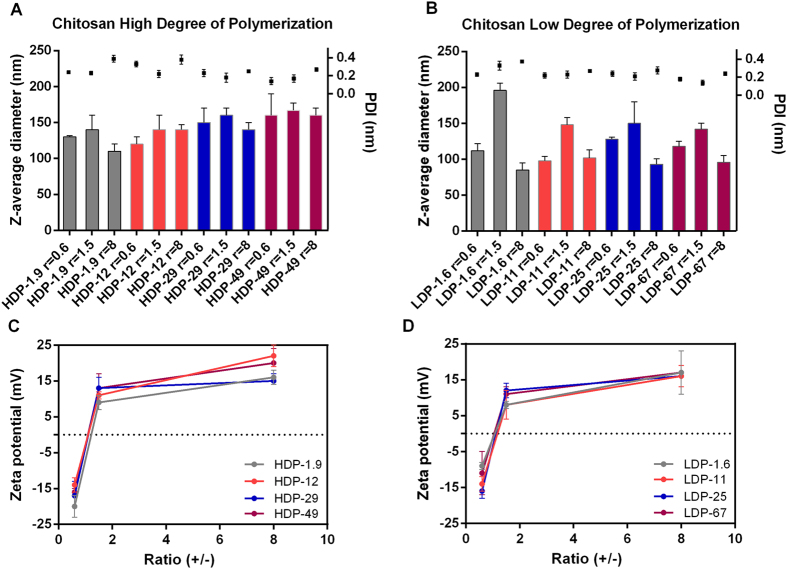
Physicochemical characteristics of self-assembled CS–miRNA complexes after incubation for 30 min at 37 °C. Z-average diameter and polydispersity index (PDI) of complexes formulated with (**A**) high degree of polymerization CS and (**B**) low degree of polymerization CS; corresponding zeta-potential values for complexes formed with (**C**) high degree of polymerization CS and (**D**) low degree of polymerization CS (*n* = 3; mean average ± SD).

The different chitosan solutions described above (made in water containing stoichiometric amounts of HCl, hence bearing a net positive charge) were mixed with a constant amount (0.05 nmol) of miRNA (hence bearing a net negative charge) to generate CS–miRNA complexes with charge ratios (+/−) in the range of 0.6–8. The resulting complexes were characterized in terms of their average diameter and zeta potential (Fig. 2). The average diameter of the complexes was ~80–190 nm although the complexes containing low-DP chitosans were marginally smaller than those containing the high-DP chitosans due to the shorter chains. The size of the complexes containing high-DP chitosans increased with increasing DA at the same charge ratio, but this phenomenon was not observed for the complexes containing low-DP chitosans. Both types of complexes increased in size as they approached the neutrality point (+/− = 1.5) where charge compensation occurs, but became smaller at higher concentrations of chitosans (Fig. 2A,B). The zeta potential varied from −20 to +20 mV for complexes containing the high-DP chitosans and from −15 to +15 mV for those containing the low-DP chitosans (Fig. 2C,D). As expected, the zeta potential always became more positive as the concentration of chitosans increased.

### Transmission electron microscopy (TEM)

**Figure 3:**
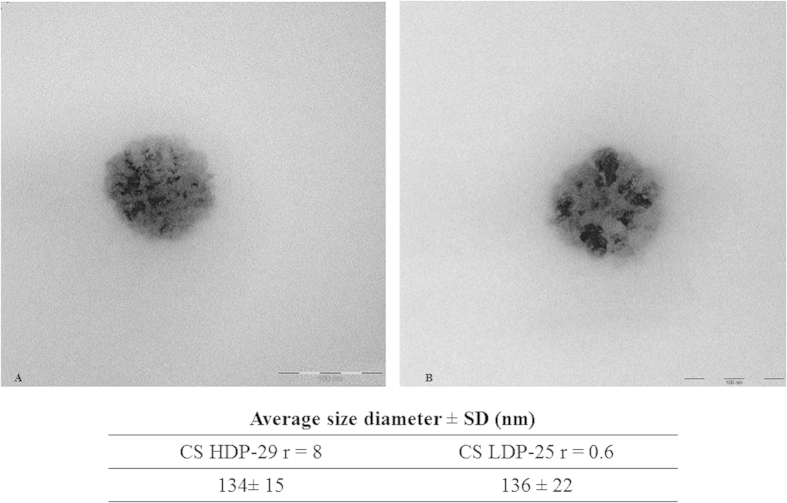
Representative TEM images of complexes containing (A) CS HDP-29 r = 8 and (B) CS LDP-25 r = 0.6 stained with uranyl acetate. The embedded table shows the measured diameter of the complexes using ImageJ v1.49n (*n* = 8; mean average ± SD).

Images of nanocomplexes containing HDP-29 or LDP-25 and miRNA were recorded by TEM and analysed in terms of particle morphology, size, and surface topology. Figure 3 shows representative images of these CS-miRNA nanocomplexes formulated at two different ratios.

The complexes were spherical in shape with a heterogeneous structure, as previously reported for 44 kDa chitosans^30^. The mean diameters of the CS-miRNA complexes determined from an average of eight TEM images using ImageJ v1.49n are reported in the table embedded in Fig. 3. These sizes correlate well with those determined by dynamic light scattering.

### Binding analysis by surface plasmon resonance (SPR)

**Figure 4:**
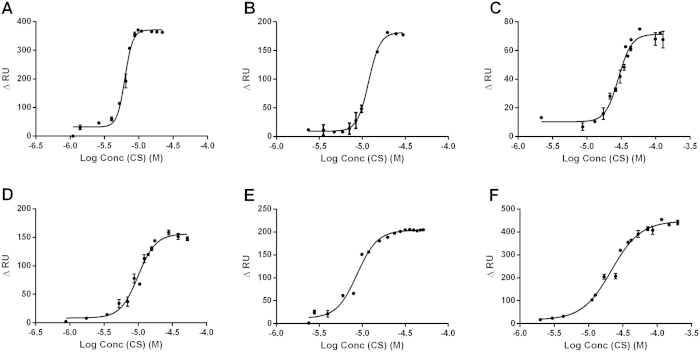
Set of saturation curves for the interaction of hsa-miR-145-5p with chitosans: (A) HDP-12, (B) HDP-29, (C) HDP-49, (D) LDP-11, (E) LDP-25 and (F) LDP-67. Binding was analysed in acetate buffer (35 mM, pH 5.1, containing 10 mM NaCl) on a streptavidin sensor chip. Increasing concentrations of chitosan were injected for 20 s at 20 μL/min until the surface was saturated. Bars represent maximum and minimum values (*n* = 2).

Biotinylated hsa-miR-145-5p was immobilized onto streptavidin sensor chips and chitosan solutions of different concentrations were passed over the flow channel allowing sensorgrams to be recorded during the interaction between chitosans and hsa-miR-145-5p (Supporting Information, S1). The response units increased significantly during the first seconds of interaction due to the formation of CS–miRNA complexes, but when a certain amount of chitosan had been added, a steady state was achieved in which the number of associating and dissociating CS–miRNA complexes is equal. The RU values in the equilibrium state were plotted against the logarithm of the corresponding chitosan concentrations. The equilibrium dissociation constants (K_D_) were extracted from these saturation curves by nonlinear regression (Fig. 4) using a sigmoidal dose response (variable slope) model. The K_D_ values are listed in Table 2.

A rapid increase in RU during the association phase was observed for both the high and low molecular weight chitosans as long as the DA was low. However, complexes containing chitosans with a higher DA were characterized by a shallower slope representing kinetically slower association. A similar trend was observed for the K_D_ values: complexes containing high-DA chitosan showed higher K_D_ values and were, therefore, significantly less stable. Taking the 95% confidence intervals into consideration, a significant difference between the K_D_ values at varying DAs was only observed for the high DP chitosans (HDP 12–49). In the case of the low-DP chitosans the corresponding complexes of LDP-11 and LDP-25 exhibited comparable K_D_ values. However, the CS–miRNA complex of LDP-67 is characterized by a significantly higher dissociation constant in comparison to the latter two (Table 2). The Hill coefficients for all complexes were greater than 1 indicating positive cooperative binding events. For high-DP chitosans the Hill coefficient significantly declined as the DA increased.

### Conformational analysis by circular dichroism spectroscopy (CD)

**Figure 5:**
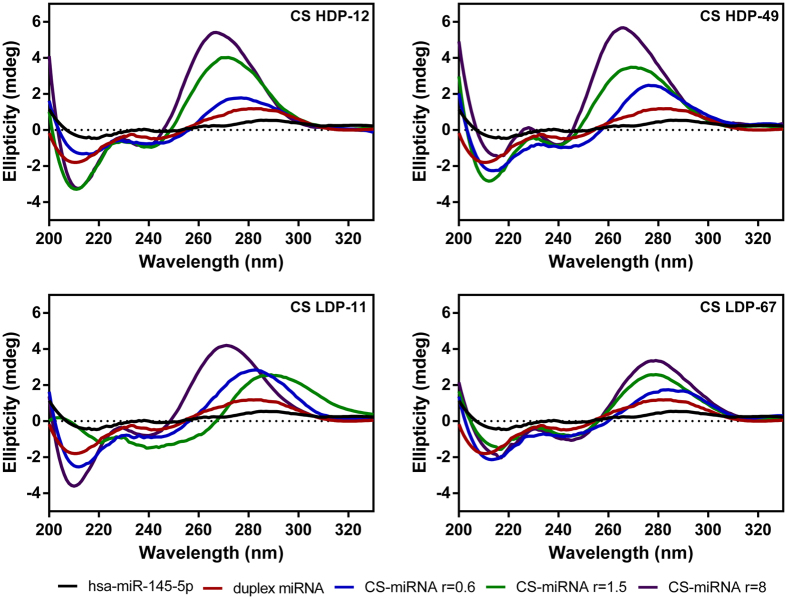
Representative CD spectra of single-stranded oligonucleotide hsa-miR-145-5p, double-stranded miRNA and different CS–miRNA complexes with (+/−) charge ratios of 0.6, 1.5 and 8. The concentration of miRNA was maintained at a constant 5 μM in all experiments. Spectra were obtained by subtracting the effect of the buffer and chitosan.

CD spectroscopy is sensitive to conformational changes in chiral asymmetric structures. Thereby changes in the CD spectra of polynucleotides are mostly dependent on the sequence of bases and the stacking geometry^31^,^32^. This method is ideal for the analysis of structural changes in RNA duplexes caused by electrostatic interactions with polysaccharides. We acquired CD spectra for single-stranded RNA (hsa-miR-145-5p), duplex miRNA-145 and complexes formed with chitosans over a range of (+/−) charge ratios (Fig. 5).

There was no absorption band in the CD spectrum for single-stranded RNA. In contrast, CD spectra for double-stranded miRNA revealed the presence of a positive band (maximum at λ = 280 nm), a negative band (minimum at λ = 210 nm), and zero CD at λ = 300 nm and beyond. These features are characteristic of the right handed A-form of double-stranded RNA^33^. The CD spectra of the double-stranded miRNA was shifted in the positive exciton band of the long-wavelength component (λ = 270 nm) following the addition of chitosans even for complexes that were deficient in chitosans (+/− = 0.6). For complexes containing excess chitosans, the leftward shift was more pronounced in all CD spectra, reflecting an increase in the positive exciton band of the long-wavelength component (λ = 270 nm). The addition of chitosans had no clear effect on the negative exciton band (λ = 210 nm). The conformational changes observed by CD spectroscopy did not appear to be influenced by the DA and DP of the chitosans, only by the (+/−) charge ratio.

### Cytotoxicity of CS–miRNA-145 towards MCF-7 breast cancer cells

The influence of nanomaterials on cell viability depends on the physical and chemical parameters of particles (i.e. size, charge, morphology and chemical composition) and the exposure conditions (i.e. cell type and density, particle concentration, medium composition, temperature, and exposure time). Chitosans with different characteristics generally show excellent biocompatibility^34^,^35^. However, chitosan–siRNA complexes with 50–70 excess positive charges caused 20–40% cytotoxicity when presented to H1299 cells^36^ and 30–40% cytotoxicity when presented to NIH 3T3 cells^37^. It was therefore necessary to monitor the potential cytotoxicity of chitosan–miRNA-145 complexes towards MCF-7 breast cancer cells.

**Figure 6:**
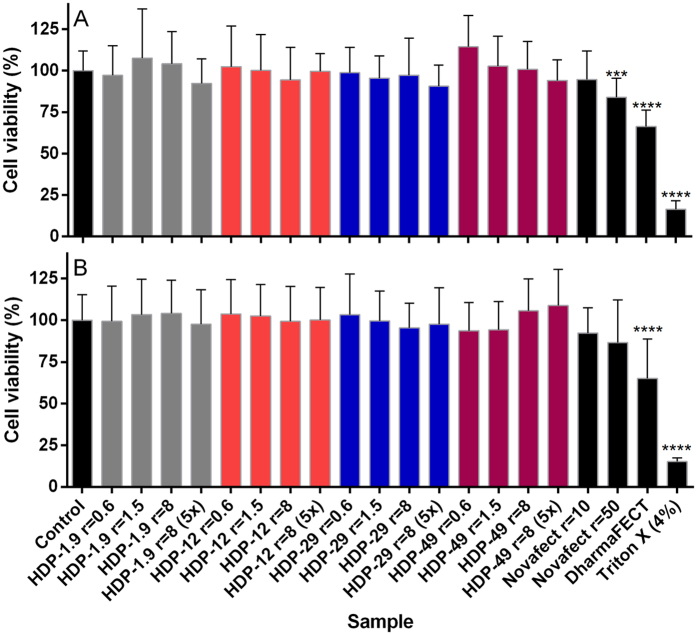
Viability of MCF-7 cells determined by MTT assays following incubation for 6 (A) and 24 h (B) with chitosan–miRNA complexes comprising CS of high degree of polymerization in RPMI minimal medium. Cell viability was expressed relative to untreated cells. Positive controls were cells treated with DharmaFECT (0.2 µL/well), Novafect O 25 and Triton X-100. The concentration of miRNA was constant (1x = 0.002 nmol/well). Data represent mean values (±SD) of three independent biological experiments and eight technical replicates. Statistical comparisons were between each treatment and the control of untreated cells using non-parametric Kruskal-Wallis test (***p ≤ 0.001; **** p ≤ 0.0001).

We evaluated the influence of CS–miRNA complexes by incubating MCF-7 breast cancer cells with high-molecular-weight complexes for 6 and 24 h at different concentrations. Three biological indep-endent experiments were performed, followed by MTT assays to determine mitochondrial dehydrogenase activity as a marker of cell viability. There was no evidence of cytotoxicity regardless of the chitosan/miRNA ratio or DA when cells were exposed for 6 or 24 h to complexes at concentrations appropriate for transfection (Fig. 6). The commercial transfection reagent DharmaFECT showed the highest statistical significant cytotoxicity both at 6 and 24 h (~65% cell viability; p ≤ 0.0001).

### Cellular uptake of CS–miRNA complexes probed by confocal laser scanning microscopy

**Figure 7:**
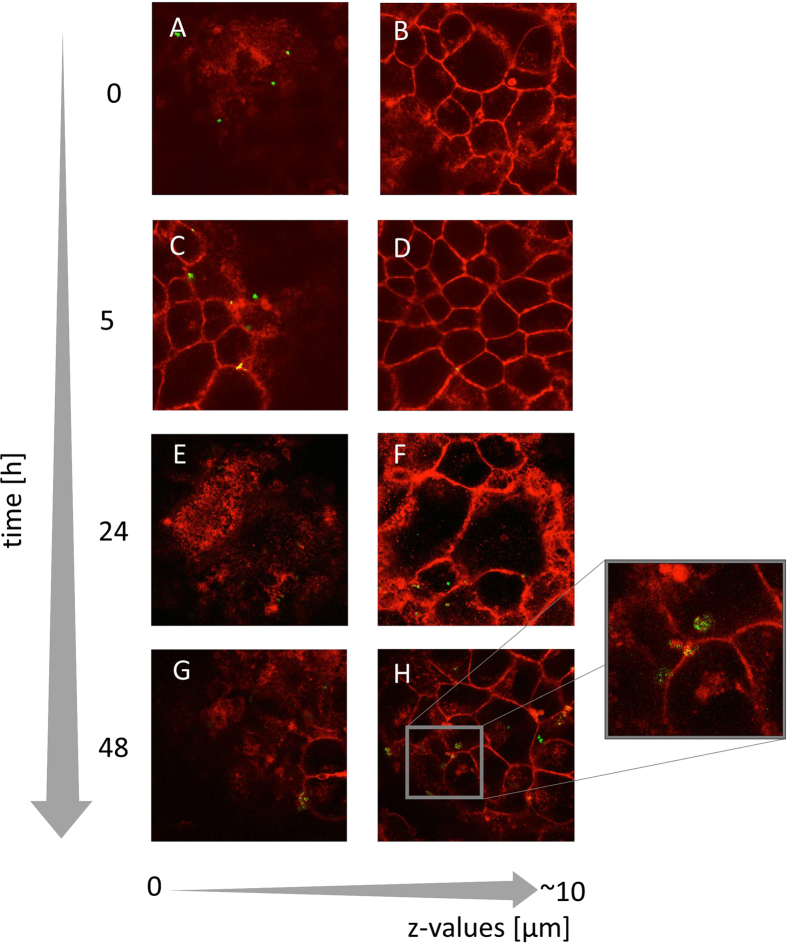
Uptake of CS HDP-12–miRNA complexes into MCF-7 cells observed by confocal laser scanning microscopy. Horizontal axis: optical sections at increasing height (z-values) Vertical axis: incubation times: 0, 5, 24 and 48 h. Red fluorescent staining = CellMask Deep Red membrane staining, Green fluorescent staining = CS HDP-12–miRNA complexes labelled with 6-FAM-hsa-miR-5p.

CLSM was used to investigate the uptake of CS HDP-12-miRNA complexes with a (+/−) charge ratio of 1.5 into MCF-7 breast cancer cells. The cell membranes were stained with CellMask Deep Red and the CS–miRNA complexes were formed with fluorescence-labelled miRNA-145 (Fig. 7).

The CLSM images revealed that most complexes were located in the medium surrounding the cells immediately after application (time zero-Fig. 7A) and no complexes were found at greater depth (Δz = 10 μm) (time zero-Fig. 7B). After 5 h of incubation the complexes persisted on the upper surface of the membrane, diagnostic of the interaction with the negatively charged surface (Fig. 7C). The complexes were still not visible at greater depth (Fig. 7D). However, after 24 h few complexes remained on the upper surface of the cell membrane (Fig. 7E) and were instead mostly localized at Δz = 10 μm, suggesting they were present in the cytoplasm; this result was more evident after 48 h of incubation, where a greater amount of internalized complexes was observed (Fig. 7H). The uptake mechanism appeared to be endocytosis, and intracellular vesicles containing CS–miRNA complexes can be seen in the enlarged image in Figure 7. However, these images offer only a qualitative evidence of the internalization process over time. Quantitative transfection efficiency was further evaluated by quantitative RT-PCR assay. Future mechanistic studies on cellular uptake and trafficking are needed.

### Transfection of MCF-7 cells with CS–miRNA-145 nanocomplexes

**Figure 8:**
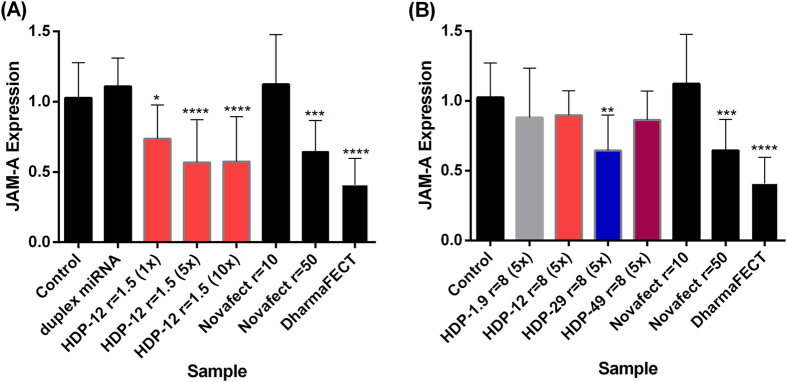
Transfection efficiency expressed as downregulation of *JAM-A* mRNA. (**A**) Complexes containing CS HDP-12 at (+/−) charge ratio = 1.5; (**B**) Complexes containing CS HDP-1.9, HDP-12, HDP-29 and HDP-49 at (+/−) charge ratio = 8. Duplex miRNA (dose 1x = 0.05 nmol/well), DharmaFECT (5 µL/well) and Novafect O 25 were used as controls. Data represent mean values (±SD) of three independent biological experiments and three technical replicates. Statistical comparisons were between each treatment and the control of untreated cells using non-parametric Kruskal-Wallis test (***p < 0.001; ****p < 0.0001).

The principal aim of this investigation was the development of a non-viral vector system based on chitosan nanoparticles that was suitable for the delivery of functional miRNAs to cancer cells using miRNA-145 as a case study. Therefore, we determined the *in vitro* efficiency of transfection with CS–miRNA-145 complexes by measuring the biological function of miRNA-145 after delivery. One of the biological functions of miRNA-145 is to downregulate junction adhesion molecule A (*JAM-A*) mRNA, which can, therefore, be used as a marker for miRNA-145 transfection efficiency^38^,^39^. We measured the level of *JAM-A* mRNA relative to a rRNA standard using the TaqMan-based quantitative RT-PCR assay, which can also detect dose-dependent silencing effects. We evaluated several scenarios using the nanocomplexes at different concentrations, (+/−) charge ratios and with different transfection reagents. Figure 8A shows the downregulation of *JAM-A* mRNA following transfection with miRNA-145 in a complex with HDP chitosan (1.5 charge ratio) at different concentrations, revealing a dose-dependent effect. There was a slight significant reduction in the abundance of *JAM-A* mRNA at the standard concentration (1x) of 0.05 nmol per well (p ≤ 0.01) but a greater significant effect at the 5x and 10x doses of 0.25 and 0.5 nmol per well, respectively (p ≤ 0.0001), when compared to the control of non-transfected cells. Both treatments caused ~55% reduction in the amount of *JAM-A* mRNA. Pure duplex miRNA-145 was used as a negative control and there was no impact on *JAM-A* mRNA detectable, suggesting that the miRNA was degraded in the medium and/or unable to cross the plasma membrane, perhaps reflecting the mutual negative charges. The transfection reagent DharmaFECT was used as a positive control, and likewise achieved a significant ~50% reduction (p ≤ 0.0001) in the level of *JAM-A* mRNA. Although this reagent shows similar transfection efficiency as chitosan complexes *in vitro* it is not recommended for *in vivo* gene delivery due to its cytotoxicity. In turn, Novafect O 25, a commercially available reagent based on chitosan, was also used as a positive control. We tested two different (+/−) charge ratios (10 and 50), while maintaining a constant quantity of miRNA. There was no significant reduction in *JAM-A* mRNA levels when cells were transfected at a (+/−) charge ratio of 10. Transfection at a (+/−) charge ratio of 50 led to a significant reduction of less than 50% (p ≤ 0.0001), thus confirming that excess of chitosan increases the transfection efficiency.

We also tested CS complexes with higher (+/−) charge ratios because better transfection efficiencies have been reported, reflecting the larger number of sites available for interaction with negative charges on the cell membrane. Additionally, a higher CS content may also help in the mechanism of gene release due to the proton sponge effect^10^. However, we found no significant improvement over the complexes with a charge ratio of 1.5 (Fig. 8). In addition it was possible to observe a significant effect (p ≤ 0.001) of the DA on the transfection efficiency, showing better results for CS with DA = 29% (Fig. 8B).

## Discussion

Efficient gene delivery is an important challenge in gene therapy because nucleic acids are negatively charged and hydrophilic. These characteristics prevent them from entering cells by passive diffusion. To find a safe and efficient non-viral vector for miRNA delivery, we investigated the interaction between chitosans and miRNA and the influence of the resulting CS–miRNA complexes on the uptake of miRNA into MCF-7 cells using *JAM-A* mRNA as a marker to monitor transfection efficiency^38^,^39^. As downregulation of *JAM-A* by miRNA-145 inhibits breast cancer and endometriotic cell invasiveness^38^,^39^, the successful application of the CS–miRNA-145 complexes marks this reagent as a potential candidate for novel antimetastatic therapeutic applications.

The characteristics of chitosan must be selected carefully to achieve the optimal particle size of CS–miRNA complexes because transfection efficiency is highly dependent on chitosan properties^10^. We found that all prepared complexes had a mean diameter of less than 190 nm, as previously reported for chitosan complexes with plasmid DNA and siRNA^14^,^30^,^40^, making them suitable for uptake by endocytosis. Chitosans with molecular weights of 25–50 kDa have been shown to bind and protect siRNAs completely from enzyme degradation, suggesting that CS-HDP used in our studies may behave in a similar fashion in complexes with miRNA^41^.

Larger complexes were also found to be produced by chitosans with higher DAs, reflecting the presence of additional hydrophobic domains that were unable to form electrostatic bonds with miRNA. However, complexes with CS of higher DAs are expected to be more flexible^42^,^43^, and can, therefore, adopt molecular configurations that are complementary to the miRNA, thereby protecting the nucleic acid from degradation. This combination of properties suggests that miRNA remains stable within high-DA chitosan complexes due to the conformational flexibility of the carrier, but is released following uptake into the cell due to the weak interactions between the two components of the complex. The latter is essential for effective gene therapy, so we investigated the binding affinity between miRNA and different chitosans to determine the mechanism of interaction and select a promising candidate for further experiments.

To the best of our knowledge, this is the first time that the interaction between miRNA and chitosans has been quantified by SPR spectroscopy. Complexes with higher DAs exhibited increased K_D_ values (Table 2). This finding reflects the effect of the charge ratio in the complexes, i.e. greater amount of polymer is required to provide the same number of protonated glucosamine units in order to saturate the system. This behaviour is congruent with previous reports using isothermal titration calorimetry to study chitosan–pDNA interactions, where it was exhibited that the DA and the molecular weight have an influence on the structure and stability of CS–pDNA complexes^44^. In this case it was reported for 80 kDa chitosans that increasing DA leads to lower binding affinities and greater stoichiometry of binding. This was explained as a result of electrostatic interactions between oppositely charged groups that determine the binding characteristics. Hence, decreasing DA of chitosans (i.e., increasing charge density) implies that fewer chitosan chains are required to reach saturation of DNA binding sites^44^. The fact that the values of the Hill coefficient varied from 2.0 to 8.0 for all CS–miRNA systems is consistent with positive cooperative binding. It is well known that polyelectrolyte complexes have in general a cooperative nature. This has been confirmed in chitosan based polyelectrolyte complexes with carrageenans forming helices^45^. Taken together, these SPR findings anticipate that higher-DA chitosans could release the miRNA into the cytoplasm more efficiently than low-DA chitosans due to the lower stability of the complexes.

The pristine miRNA was shown by CD spectroscopy to adopt the A-form conformation^33^, which confirmed the success of hybridization and the formation of a duplex between the two oligonucleotide strands. The change in the CD spectrum during the formation of CS–miRNA complexes indicated a concentration-dependent increase in base stacking. The electronic distribution of bases in dsRNA makes them hydrophobic, so they tend to stack in the presence of hydrogen-bonding solvents in order to minimize the π-electron surface area exposed to the solvent. However, hydrophilic groups such as NH, NH_2_ and CO tend to become orientated to the edges of the bases and favour interactions with hydrogen-bonding solvents. The helical structure forms due to the base stacking interactions, reflecting the hydrophobic planes, hydrophilic edges and charge-charge interactions^32^. The interaction with chitosans appears to enhance base stacking in all the complexes. The electronic transitions of the chromophore bases are in close proximity, yielding CD spectra with more intense bands. This suggests that all the chitosans were able to interact with miRNA, leading to changes in the backbone conformation and base stacking. Because the interactions were driven by electrostatic forces, the effect on the miRNA conformation was dependent on the (+/−) charge ratio as confirmed by the CD spectra. The molecular weight and DA had no substantial effect on the conformation and the most important property was the number of amino groups on the chitosan polymer.

Chitosans are generally biocompatible and several studies have confirmed that chitosan–oligonuc-leotide complexes show low cytotoxicity^15^,^46^. Previous reports have demonstrated the cytotoxicity of high molecular weight chitosans^47^. However, our MTT assay showed no evidence of cytotoxicity following exposure for 6 and 24 h even with complexes containing the highest concentrations of chitosan. The internalization of the complexes was investigated by CLSM using a fluorescent marker, suggesting that the complexes interact with the membrane and induces the formation of endosomes, as we previously reported^6^. Further cytoplasmic release is thought to be driven according to the proton sponge hypothesis, which offers a convincing explanation to the dissociation of polyplexes at the intracellular vesicles^41^.

We found that certain concentrations of chitosans with a particular (+/−) charge ratio were as efficient as commercial transfection reagents. Our combined data suggest that complexes containing low molecular weight chitosans are unsuitable for gene delivery because they are unstable in the transfection medium, and that complexes containing intermediate-DA chitosans are efficient. This may suggest that a compromise is reached between the formation of stable complexes able to interact with the cell membrane and sufficiently unstable to favour the endosomal release for reliable delivery of miRNA to the cytoplasm. In contrast, complexes containing low-DA chitosans are too stable and would not release the miRNA cargo, as indicated by the high relative *JAM-A* expression and the high affinity constants determined by fluorescence titration in an ongoing parallel study in our group^48^. Negatively charged complexes (i.e., (+/−) charge ratios lower than 1.0) were found unsuitable for transfection. This may be because the plasma membrane carries a similar charge and hence the interaction with the complexes is not favourable, while positively charged complexes are only efficient when they exceed a certain concentration. This led us to conclude that optimal transfection occurs when the molecular weight, DA and (+/−) charge ratios are perfectly balanced. This interpretation is in agreement with previous findings for chitosan-DNA complexes^40^.

In general, cationic non-viral vectors have been used successfully to deliver siRNA and plasmid DNA but not miRNA. We found that chitosans can be used as vectors for miRNA if the formulation parameters are carefully balanced. CD spectroscopy confirmed that the miRNA remained in the A-conformation when bound to chitosan, and we also used SPR spectroscopy for the first time to quantify the binding affinity between miRNA and chitosans with different properties. We found that ideal complexes were formed using chitosans with a molecular weight of ~40 kDa, DA of 12%, and a (+/−) charge ratio of 1.5, resulting in transfection efficiencies similar to the commercial reagents used as positive controls (DharmaFECT and Novafect O 25). We showed that the DA has an influence on the transfection efficiency for complexes with equivalent (+/−) charge ratio (8.0): more efficient downregulation of the target gene in the presence of intermediate DA (~30%). CLSM suggested that the complexes may have been taken up by the cells. However, the precise mechanisms of the intracellular release pathways remain to be fully elucidated.

Nontoxic functional nanocarriers based on miRNA delivery are revolutionizing the development of targeted cancer therapies. A robust delivery system functionalized with ligands that recognize specific receptors in tumour cell membranes will bring significant advances in the field, and chitosans appear to be versatile delivery vehicles to achieve these aims. Future studies in animal models aiming to obtain proof of principle of the *in vivo* efficacy of these systems will also be key in this regard.

## Materials and Methods

### Preparation of chitosans

The parent chitosan used to prepare the working samples was provided by Sascha Mahtani Chitosan PVT Ltd (Veraval, India; Code 113 Batch No. 17/12/04; DA = 1.5%, Mw = 543 kDa). Eight high purity biomedical-grade chitosans were produced with different molecular weights and DAs. The parent chitosan was stoichiometrically dissolved in acetic acid solution overnight at room temperature and depolymerized using sodium nitrite (forming nitrous acid *in situ* as previously reported^24^,^25^), to generate chitosans with a high degree of polymerization (HDP) and a low degree of polymerization (LDP), respectively. The pH was increased to >8 by adding ammonia and the precipitated chitosans were washed to achieve neutrality before freeze-drying. HDP and LDP chitosans were re-acetylated by dissolving them stoichiometrically in acetic acid solution to a concentration of 12.5 mg/mL, and passing them sequentially through 5, 1.2, 0.8 and 0.45 μm filters to eliminate agglomerates and reduce polydispersity. We then added acetic anhydride and 1,2-propanediol and the reaction mixtures were incubated for 2 h at room temperature. The reactions were stopped by precipitation as described above^49^. The freeze-dried samples were used for the production of nanoparticles.

### Characterization of chitosans

The relative viscosity of a series of diluted chitosan solutions of varying concentrations in 0.3 M acetic acid and 0.2 M sodium acetate was measured at 25 °C (inclination angle, 50°) using an AMVn automated rolling ball microviscosimeter (Anton Paar, Ostfildern, Germany) with a programmable tube angle based on the principle of the rolling ball time (the time required for the steel ball to roll inside a calibrated 1.6-mm diameter capillary). The average results obtained from four runs were expressed as the intrinsic viscosity [η] and as the viscosity average molecular weight (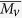) using the Mark-Houwink parameters^28^,^29^,^50^. The DA was determined by ^1^H-NMR at 300 MHz and room temperature using a Bruker AV300 NMR spectrometer (Bruker, Bremen, Germany)^26^,^27^. NMR samples (~5 mg) were dissolved in 0.5 mL of a mixture of deuterium chloride (DCl) and deuterated water (D_2_O) with a ~5% stoichiometric excess of DCl over the molar glucosamine content of the corresponding chitosan. Subsequently the sample was freeze-dried and redissolved in D_2_O for three times to remove exchangeable protons.

### Hybridization

Single-stranded microRNAs hsa-miR-145-5p (5′-GUC CAG UUU UCC CAG GAA UCC CU-3′) and hsa-miR-145-3p (5′-GGA UUC CUG GAA AUA CUG UUC-3′) were purchased from Biomers (Ulm, Germany). They were dissolved in RNase-free water to produce solutions of equal concentration (50 μM), mixed and heated to 90 °C for 4 min to remove secondary structures. The solutions were then cooled to 40 °C and incubated for 3 h to promote hybridization. The formation of double stranded miRNA-145 was analysed by 15% polyacrylamide gel electrophoresis with 1 × TAE buffer. Each lane was loaded with 5 μL of the sample (5 μM) mixed with 2 × RNA loading dye (Thermo Fischer Scientific Inc., Waltham, USA). We used as a marker a Generuler ultra low range DNA ladder (Thermo Fischer Scientific Inc., Waltham, USA) that contains 11 individual chromatography-purified DNA fragments. Electrophoresis was carried out at a constant voltage of 120 V for 90 min. The gel was stained with SYBRGold (Life Technologies, Carlsbad, CA, USA). RNA bands were visualized using a UV transilluminator (AAB Advanced American Biotechnology, CA, USA) and the gel image was captured by a video camera (Model CCD-440, AAB Advanced American Biotechnology, CA, USA).

### Formation of CS–miRNA complexes

Fully characterized chitosans were used to produce CS–miRNA polyplexes. The chitosans were dissolved in stoichiometric amounts of HCl to a stock concentration of 1 mg/mL, and then diluted with milliQ water to reach the desired amine concentration (deacetylated groups). A series of complexes was then prepared at different charge ratios (+/−, defined as the molar ratio of amine to phosphate groups) by mixing the chitosan working solutions with a constant amount of miRNA (5 μM). The mixtures were incubated for 30 min at 37 °C^11^,^40^.

### Size distribution and zeta potential of CS–miRNA complexes

To determine the hydrodynamic size and zeta potential of the above described CS–miRNA complexes, the samples were analyzed at 37 °C in a Zetasizer Nano ZS (Malvern Instruments, UK). The hydrodynamic size was determined by dynamic light scattering in 100 μL aliquots. The samples were then diluted to 1 mL and the zeta potential was measured by determining the electrophoretic mobility. All measurements were carried out in triplicate and were presented as the average of three measurements ± standard deviation.

### Transmission electron microscopy

Polyplexes were visualized by transmission electron microscopy (TEM) using 10 μL samples (5 μM in RNase-free water) diluted 1:10 RNase-free water and mixed with 10 μL 1% (w/v) uranyl acetate for negative staining. Afterwards 10 μL of the samples were deposited onto a copper grid covered with a Formvar® film. Excess liquid was blotted using filter paper. Images were captured using a Philips CM100 TEM (Eindhoven, Netherlands). Images were processed using ImageJ v1.49n to calculate the average particle diameter (n = 8).

### Surface plasmon resonance spectroscopy

Biotinylated hsa-miR-145-5p was purchased from Biomers (Ulm, Germany). All surface plasmon resonance (SPR) spectroscopy experiments were carried out using a Biacore 3000 instrument (Biacore, Uppsala, Sweden) at 20 °C. HBS-EP buffer (GE Healthcare, Uppsala, Sweden) was used as a running buffer for the immobilization procedure (immobilization buffer) and acetate buffer (35 mM sodium acetate, pH 5.1, containing 10 mM NaCl) was used as the running buffer during the measurements. All buffers were prepared in RNase-free water, sterile filtered and degassed before use. Before immobilization, the surface of the streptavidin sensor chip (GE Healthcare, Uppsala, Sweden) was primed three times with immobilization buffer and prepared by injecting 20 µL of 50 mM NaOH/1 M NaCl three times at a flow rate of 20 μL/min. The surface was washed with immobilization buffer until a stable baseline was achieved, then 70% (w/w) glycerol (BIAnormalizing solution from GE Healthcare) was injected to prevent inaccuracies caused by divergent reflection properties. After priming the system three times, 400 response units (RUs) of the single-stranded RNA (40 nM) were immobilized at a flow rate of 5 μL/min. Flow cell one was left blank as a reference. Before the first measurement, the chip was regenerated using two injections of 10 μL regeneration buffer (1 M NaCl/6 mM HCl). The immobilization buffer was then exchanged with the measurement buffer in priming steps. The sensorgrams were recorded at a flow rate of 20 μL/min. At the beginning of each cycle, the chip surface was equilibrated with measurement buffer for 3 min. Subsequently, 20 μL of each chitosan in running buffer were injected for 4 min. The dissociation phase was monitored for 5 min followed by a regeneration step with two 10 μL injections of regeneration buffer and the surface was washed with running buffer for another 5 min. The average of two measurements was used for data analysis and the RUs at equilibrium phase were evaluated by nonlinear regression analysis (GraphPad Software, San Diego, USA).

### Circular dichroism spectroscopy

Conformational changes in duplex miRNAs caused by interactions with chitosans were analysed using an AVIV 400 circular dichroism spectrophotometer (Maryland, United States). Each 100-μL microRNA sample (10 μM) was mixed with 100 μL chitosan solution in 6 mM HCl to form complexes with (+/−) charge ratios ranging from 0.6 to 8. The samples were incubated for 30 min and placed in 1 mm quartz cuvettes, and the spectra were recorded at 25 °C and a scanning speed of 200 nm/min from 320 to 200 nm, with a response time of 1 s and a bandwidth and data pitch of 1 nm. Each spectrum was accumulated from three scans.

### Cell studies

#### Metabolic capability (MTT assay)

MCF-7 cells (10,000 cells/well) were seeded in 96-well plates and incubated for 24 h at 37 °C and 5% CO_2_. The medium was removed and the cells were washed twice with RPMI serum-free medium. The complexes were prepared in RPMI serum-free medium and incubated for 30 min at 37 °C. We then added 100 μL of each sample to the cells and incubated them for 6 and 24 h at 37 °C and 5% CO_2_. Cell viability was determined by measuring dehydrogenase activity. We changed the medium and applied 100 μL of RPMI serum-free medium with 25 μL of MTT (3-(4,5-dimethylthiazol-2-yl)-2,5-diphenyltetrazolium bromide) (5 mg/mL) to each well and incubated the cells for 4 h at 37 °C and 5% CO_2_ to allow the formation of a purple formazan salt. The medium was replaced with 100 μL of dimethylsulfoxide to dissolve the formazan crystals and the plates were incubated for a further 15 min at 37 °C and 5% CO_2_ before the absorbance was measured at λ = 570 nm using a Micro Plate Reader (SAFIRE II, Tecan Group Ltd., Männedorf, Switzerland).

#### Confocal laser scanning microscopy (CLSM)

We investigated the intracellular trafficking of nanocomplexes containing 6-FAM-hsa-miR-5p (Biomers, Ulm, Germany). MCF-7 cells were cultured in 35-mm dishes with glass coverslip bottoms. The cells were transfected with 5 μmol 6-FAM-hsa-miR-5p as a complex with HDP-12 at a (+/−) charge ratio of 1.5 for 24 h. The uptake of miRNA was evaluated at 0.5, 4, 24 and 48 h after transfection using a Leica TCS SP2 mounted on a Leica DM IRES inverted microscope. At each time point, the transfection medium was removed and the cell membranes were stained with CellMask Deep Red for 10 min at 37 °C, following the manufacturer’s protocol (Thermo Fisher Scientific Inc., Waltham, USA). The cells were washed twice at 37 °C with phosphate buffered saline (PBS) containing calcium and magnesium, as provided by the manufacturer. CellMask Deep Red was visualized at excitation and emission wavelengths of 649 and 666 nm, respectively, whereas 6-FAM-hsa-miR-5p was visualized at excitation and emission wavelengths of 488 and 518 nm, respectively.

#### Transfection efficiency of CS–miRNA complexes

Transfection assays were conducted in conditions according with the standard protocol recommended for DharmaFECT. Briefly, we used a constant volume of 10 μL miRNA-145 (5 μM) per transfection, mixed with different chitosans to achieve the desired molar charge ratio. The complexes were diluted to 400 μL with RPMI minimal medium and incubated for 30 min at 37 °C. MCF-7 cells were seeded into six-well plates (250,000 cells per well) and incubated for 24 h prior to transfection. The medium was then removed and replaced with 1600 μL RPMI complete medium supplemented with 10% fetal calf serum (FCS) and added with 400 μL of each complex solution (in RPMI minimal medium). The cells were incubated with the transfection medium for 24 h at 37 °C and 5% CO_2_, replaced with RPMI complete medium containing 10% FCS and incubated for further 24 h. Control cells were incubated with medium only (1600 μL RPMI complete medium containing 10% FCS and 400 μL RPMI minimal medium). We also prepared controls comprising pristine miRNA, DharmaFECT (Thermo Fischer Scientific Inc., Waltham, USA) loaded miRNA, and Novafect O 25 (Novamatrix, Sanvika, Norway) containing miRNA at two different ratios. In these latter cases, we followed the manufacturers’ transfection protocols.

#### RNA isolation and cDNA synthesis

Total RNA was isolated using the innuPREP DNA/RNA Mini Kit (Analytik Jena AG, Jena, Germany) and the amount of purified total RNA was determined by measuring the absorbance at λ = 260 nm using an Eppendorf spectrophotometer (Hamburg, Germany). First strand cDNA synthesis was carried out using 2 μg of isolated RNA and the High Capacity cDNA Reverse Transcription Kit according to the manufacturer’s recommendations (Applied Biosystems, California, USA).

#### Quantitative TaqMan real-time PCR analysis

Transfection efficiency was evaluated by measuring the abundance of junction adhesion molecule A (*JAM-A*) mRNA, a direct target of miRNA-145, which is encoded by *F11R* gene^39^. We used cDNA corresponding to 0.05 μg total RNA as a template for PCR amplification with the ABI Master Mix and TaqMan gene expression systems. We used the Hs00170991_m1 (F11R) probe to quantify *JAM-A* mRNA levels and the Hs99999901_s1 (18S) probe to normalize target mRNA levels to the abundance of 18*S* rRNA. Quantitative real-time PCR was carried out using the ABI PRISM 7300 sequence detection system (Applied Biosystems, Foster City, CA, USA) with default thermal cycling conditions. The relative *JAM-A* expression level was calculated using the corresponding Ct values normalized against the values recorded in the control cells.

### Data analysis

Statistical analysis was carried out using GraphPad Software Prism v6 (San Diego, USA). All experiments were statistically analysed using non-parametric tests using the Kruskal-Wallis test to compare non-parametric data. All biological experiments were conducted at least in triplicate and technical replicates varied from 3 to 8. All the treatments were independently compared to the control of cells incubated only with medium. Statistical significant differences were evaluated by the p-values.

## Additional Information

**How to cite this article**: Santos-Carballal, B. *et al.* Physicochemical and biological characterization of chitosan-microRNA nanocomplexes for gene delivery to MCF-7 breast cancer cells. *Sci. Rep.* **5**, 13567; doi: 10.1038/srep13567 (2015).

## Supplementary Material

Supplementary Information

## Figures and Tables

**Table 1:** Physicochemical characteristics of prepared chitosans: degree of acetylation (DA) calculated by ^1^H-NMR, intrinsic viscosity ([η]) determined by viscosimetry (0.3 M acetic acid/0.2 M sodium acetate at 25°C) and viscosity average molecular weight (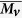).^29^

Chitosan Sample	DA (%)	[η] (mL/g)	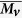 (Da)
HDP-1.9	1.9	259	26100
HDP-12	12	248	25500
HDP-29	29	215	20200
HDP-49	49	215	18000
LDP-1.6	1.6	24	1300
LDP-11	11	21	1200
LDP-25	25	21	1140
LDP-67	67	29	1950

**Table 2:** Binding parameters of different chitosans in complexes with hsa-miR-145-5p calculated from the fit sigmoidal dose response (variable slope) model using GraphPad Prism v6.

Chitosan Sample	K_D_ (μM)	95% CI [K_D_] (μM)	Hill coefficient ± SD	R^2^
HDP-12	6.276	5.932–6.640	8 ± 1	0.9892
HDP-29	11.84	11.40–12.30	6.5 ± 0.4	0.9986
HDP-49	28.89	26.28–31.76	4.6 ± 0.7	0.9762
LDP-11	9.982	8.774–11.36	3.4 ± 0.6	0.9784
LDP-25	8.704	7.824–9.684	3.7 ± 0.6	0.9834
LDP-67	21.52	18.51–25.01	2.0 ± 0.3	0.9867
