# Supplementary material for: Physicochemical and biological characterization of chitosan-microRNA nanocomplexes for gene delivery to MCF-7 breast cancer cells
Source: Sci Rep. 2015 Sep 1;5:13567. doi: 10.1038/srep13567 (PMC4555168; doi:10.1038/srep13567)
Supplement: Supplementary Information [file srep13567-s1.pdf]

Physicochemical and biological characterization of chitosan-microRNA nanocomplexes for gene delivery to MCF-7 breast cancer cells

Santos-Carballal B.[1], Aaldering L.J.[1], Ritzefeld M.[2], Pereira S.[1], Sewald N.[2], Moerschbacher B.M.[1], Götte M.[3*], Goycoolea F.M.[1*]

[1] Institute of Plant Biology and Biotechnology (IBBP), University of Münster, Schlossgarten 3, D-48149 Münster, Germany

[2] Organic and Bioorganic Chemistry, Bielefeld University, Universitaetsstrasse 25, D-33615 Bielefeld, Germany

[3] Department of Gynecology and Obstetrics, University of Münster, Albert-Schweitzer-Campus 1, D-48149 Münster, Germany

Corresponding authors:

Martin Götte (mgotte@uni-muenster.de) Fax: +49 251 83-55928

Francisco M. Goycoolea (goycoole@uni-muenster.de) Fax: +49 251 83-28371

Present address Markus Ritzefeld: Department of Chemistry, Imperial College London, South Kensington Campus, SW7 2AZ, London, UK

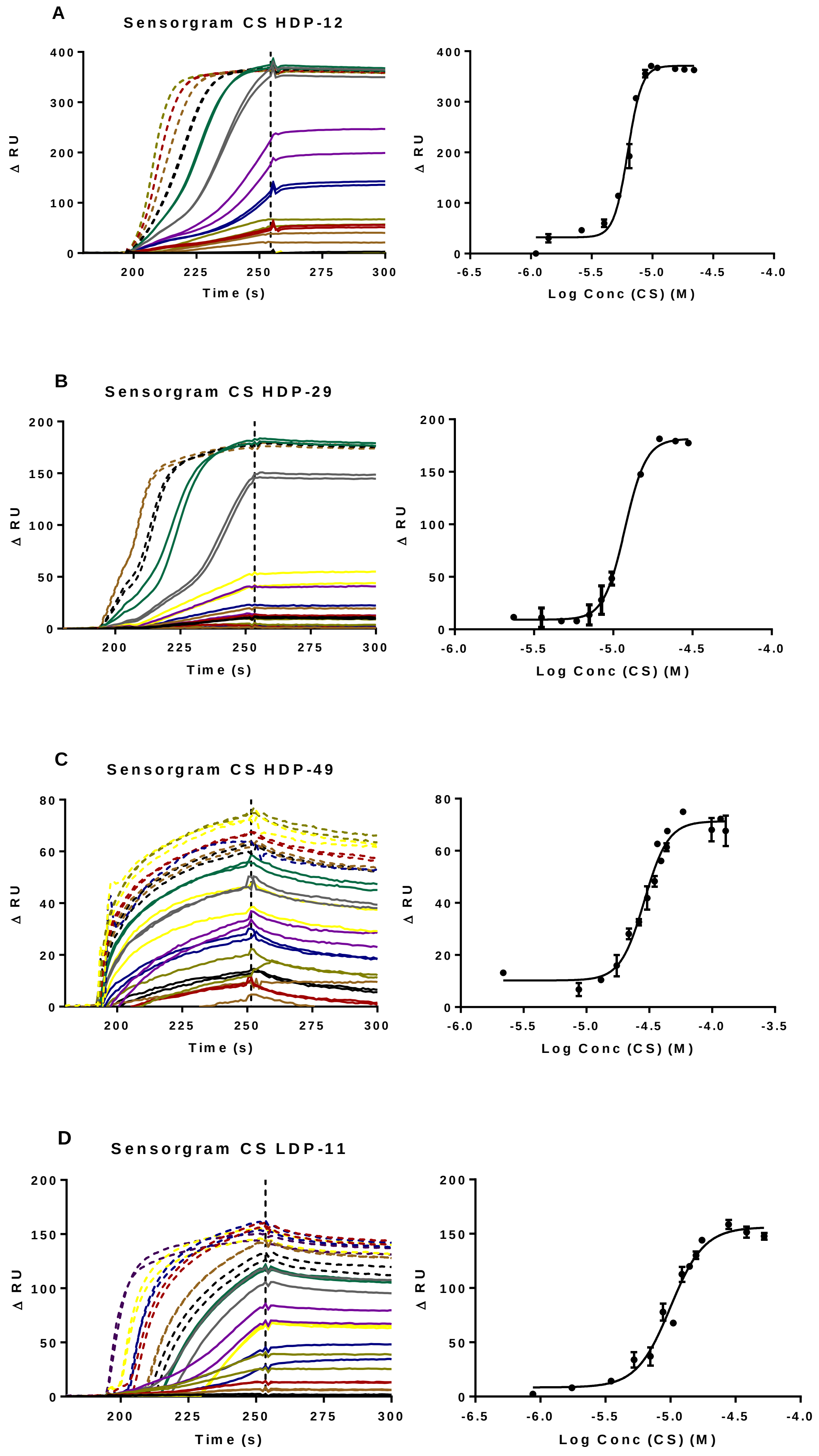
A
Sensorgram CS HDP-12
Δ RU
Time (s)
Log Conc (CS) (M)
B
Sensorgram CS HDP-29
Δ RU
Time (s)
Log Conc (CS) (M)
C
Sensorgram CS HDP-49
Δ RU
Time (s)
Log Conc (CS) (M)
D
Sensorgram CS LDP-11
Δ RU
Time (s)
Log Conc (CS) (M)

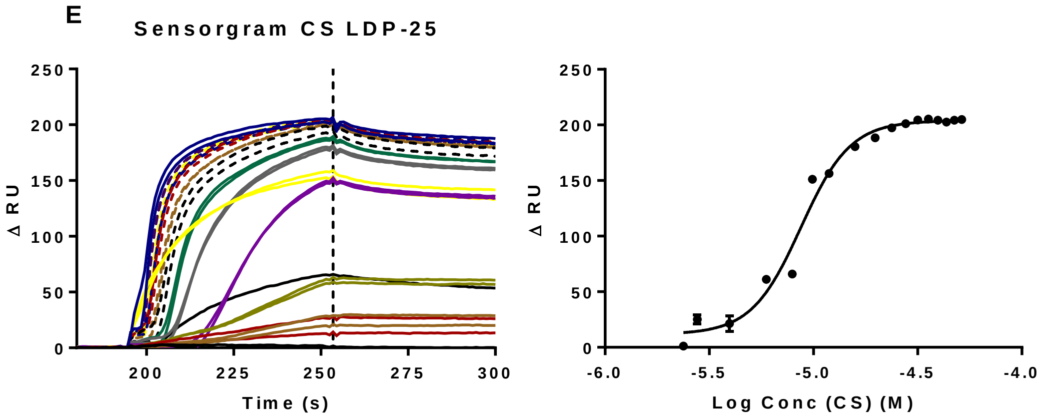

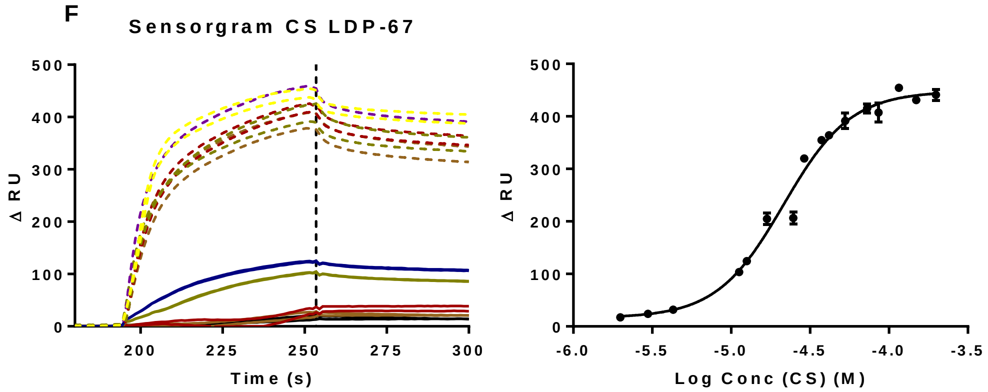

S1. Sensorgrams of different chitosans interacting with hsa-miR-5p on streptavidin sensor chip. Binding was analyzed in acetate buffer (pH=5.1) containing 10mM NaCl. Increasing concentrations of chitosan were injected over the surfaces for 20 s at 20 μL/min until the surface was saturated.

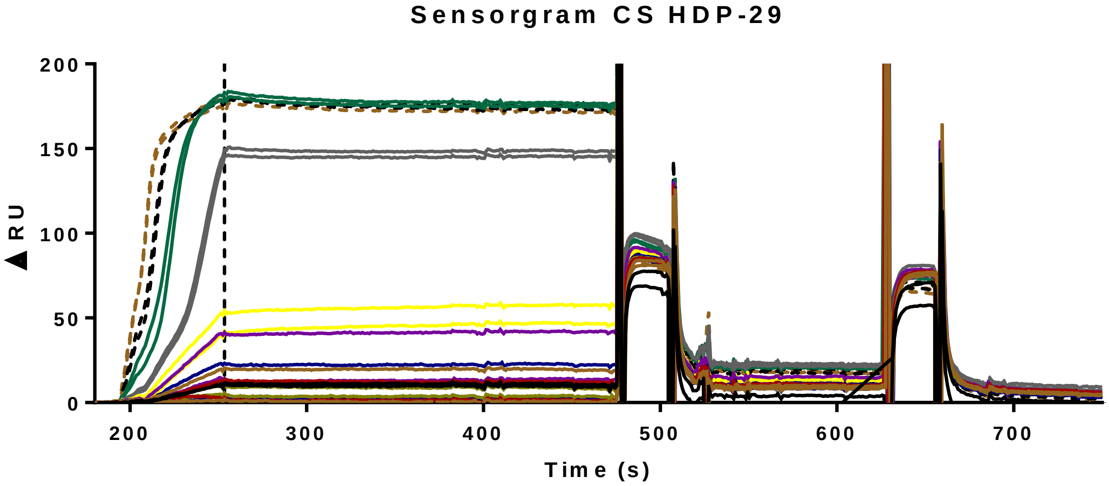

S2. Complete sensorgram with regeneration phases of CS HDP-29 interacting with hsa-miR-5p on streptavidin sensor chip. Binding was analyzed in acetate buffer (pH=5.1) containing 10mM NaCl. Increasing concentrations of chitosan were injected over the surfaces for 20 s at 20 µL/min until the surface was saturated.
